# The feasibility and potential benefits of administering adjuvant chemotherapy in resected pancreatic cancer patients unable to promptly remove intraperitoneal drainage post-surgery: a retrospective cohort study

**DOI:** 10.1186/s12885-025-14262-1

**Published:** 2025-05-20

**Authors:** Dong Xu, Nan Lv, Qianqian Wang, Yang Wu, Kai Zhang, Yi Miao, Jishu Wei, Min Tu, Kuirong Jiang

**Affiliations:** Pancreas Center, The First Affiliated Hospital of Nanjing Medical University (Jiangsu Provincial People’s Hospital), Pancreas Research Institute, Nanjing Medical University, Nanjing, Jiangsu Province 210029 China

**Keywords:** Pancreatic cancer, Postoperative pancreatic fistula, Intraperitoneal drainage, Adjuvant chemotherapy, Recurrence-free survival

## Abstract

**Objective:**

Pancreatectomy remains associated with a high incidence of complications. In certain cases, patients with pancreatic ductal adenocarcinoma (PDAC) face challenges in removing intraperitoneal drainage after surgery, leading to potential delays in the administration of adjuvant chemotherapy (AC) and potentially impacting survival outcomes. The objective of this study was to evaluate the feasibility and potential benefits of AC in PDAC patients who are unable to remove intraperitoneal drainage over 30 days.

**Methods:**

Between January 2021 and December 2022, a total of 220 patients with resected PDAC received AC at our center. Among them, 84 patients experienced persistent intraperitoneal drainage lasting more than 30 days postoperatively. Of these, 38 patients (45.2%) initiated AC despite the ongoing presence of drainage and were classified as the AC(d+) group, while the remaining 46 patients (54.8%) began AC only after successful drainage removal, and were categorized as the AC(d−) group. The other 136 patients, who underwent prompt removal of intraperitoneal drainage, were assigned to the AC(pr) group. Baseline information, surgery-related outcomes, and chemotherapy-related adverse events were collected and compared between the two groups, and factors that affected recurrence-free survival (RFS) were also analysed.

**Results:**

Of the 220 patients included in the study, 107 (48.7%) experienced grade 3–4 chemotherapy-related adverse events. The interval from surgery to the initiation of AC was similar between the AC(d+) and AC(pr) groups (50 vs. 57 days, *P* = 0.108). However, it was significantly shorter in the AC(d+) group compared to the AC(d−) group (50 vs. 61 days, *P* = 0.015). Notably, no additional chemotherapy-related adverse events were observed in the AC(d+) group compared to either the AC(d−) or AC(pr) groups. The estimated 1-year and 2-year survival rates were 85.6% and 60.5%, respectively, for the AC(d−) group, and 95.8% and 61.0% for the AC(d+) group. In the AC(pr) group, the corresponding survival rates were 89.1% and 64.0%. Cox multivariate regression analysis demonstrated that tumour grade differentiation, completed six cycles of therapy, the interval from surgery to the initiation of AC and resection margins were independent factors affecting RFS.

**Conclusion:**

Administering AC was safe for patients who underwent resection for PDAC and encountered challenges in the prompt removal of intraperitoneal drainage beyond 30 days post-surgery. The proactive management of preventing delays in chemotherapy administration could reduce the early recurrence risk in this particular patient cohort.

## Introduction

Pancreatic ductal adenocarcinoma (PDAC) is a highly lethal cancer with a dismal 5-year survival rate of less than 11% [1]. Despite the typically advanced stage upon diagnosis, patients undergoing primary tumour resection achieve the best outcomes [2]. The prevailing incidence of complications post pancreatectomy remains conspicuously high, underscored by the rate of occurrence of postoperative pancreatic fistula spanning from 5–26% 3]. This also contributes to the situation where numerous patients undergoing pancreatic cancer resection are unable to promptly remove the intraperitoneal drainage.

Adjuvant chemotherapy (AC) following potentially curative resection is crucial in preventing early recurrence and improving overall survival (OS) [2]. Based on the research from ESPAC-3, the ability to complete the full course of chemotherapy is more important than the timing of its initiation for the majority of pancreatic cancer patients [4]. However, for certain patients, such as those who were unable to complete the full course of chemotherapy as observed in the ESPAC-3 study, 4]. or patients with R1/R2 resections, or individuals who test positive for postoperative circulating tumor cells, early chemotherapy might confer survival benefits. 4, 5].

In patients with complications, however, intraperitoneal drainage may need to be retained for more than 8 weeks, and in some cases up to 12 weeks, leading to a prolonged delay in the initiation of AC [6]. Additionally, it is essential to underscore that the patient’s eligibility for AC primarily depends on their performance status, good biliary drainage, and adequate nutritional intake [7]. Even in the presence of intraperitoneal drainage, provided that drainage is unobstructed and that these factors are met, AC may still be possible.

Thus, the objective of this study was to evaluate the feasibility and potential benefits of AC in pancreatic cancer patients who are unable to remove intraperitoneal drainage for more than 30 days following surgery, subject to meeting other appropriate criteria.

## Materials and methods

### Patient cohort

There was performed a retrospective review of all patients who underwent radical resection for pancreatic cancer at the Pancreatic Centre of the First Affiliated Hospital of Nanjing Medical University between January 2021 and December 2022. The inclusion criteria comprised: (1) pathological confirmation of PDAC, (2)undergone radical resection. The exclusion criteria were as follows: (1) concurrent presence of other malignancies, (2) lack of AC after radical resection, and (3) major information missing. All patients signed an informed consent form, which was approved by the Ethics Committee of the First Affiliated Hospital of Nanjing Medical University.

A total of 220 patients with PDAC who underwent radical resection and received AC were included in the study. Among them, 84 patients experienced persistent intraperitoneal drainage lasting more than 30 days postoperatively. Of these, 38 patients (45.2%) initiated AC despite the ongoing presence of drainage and were classified as the AC(d+) group, while the remaining 46 patients (54.8%) began AC only after successful removal of the drainage, and were categorized as the AC(d-) group. The other 136 patients, who underwent prompt removal of intraperitoneal drainage, were assigned to the AC(pr) group.

### Surgical procedure and intraperitoneal drainage placement

(1) Pancreaticoduodenectomy (PD): Standard or extended pancreaticoduodenectomy is conducted, followed by reconstruction of the digestive tract via the Child method. A modified single-layer pancreatic duct–mucosa anastomosis is utilised for pancreatico-jejunal anastomosis. (2) Distal pancreatectomy (DP): Standard or extended distal pancreatectomy is conducted, comprising resection of the pancreatic tail, spleen, and splenic vessels, lymph node dissection, and closure of the pancreatic remnant by means of isolated ligation of the pancreatic duct followed by intermittent suture closure of the pancreatic remnant or a linear cutting stapler. (3) Following the conclusion of PD or DP, it is customary to insert 2–3 intraperitoneal drainage tubes in the vicinity of the pancreatico-jejunal anastomosis site or the pancreatic remnant for the purpose of drainage.

### Drainage removal criteria

Amylase in the abdominal drainage fluid is tested on postoperative days 1, 3, and 5, and the characteristics of the drainage fluid are observed to determine the presence of pancreatic fistula, biliary fistula, or intestinal fistula. If no such fistulas are identified as described above, and the drainage fluid appears clear and resembles ascites, the drainage tube is typically removed between postoperative days 5–7.

If the fistulas are present, but the patient: (I) Remains in generally good condition with no fever; (II) Displays relatively normal infection markers (WBC, PCT, CRP, etc.); (III) Exhibits no abdominal fluid accumulation upon ultrasound/CT examination, it may be considered to discharge the patient without drainage removal, followed by regular follow-up appointments.

After discharge with a drain in place, if there is minimal to no fluid output for 2–3 days (< 15-20 ml/day), and the patient continues to meet criteria I, II, and III, it may be appropriate to consider the removal of drainage.

### Adjuvant regimen

The chemotherapy protocol comprises first-line AC regimens, which consist of the mFOLFIRINOX regimen (irinotecan, oxaliplatin, calcium folinate, and 5-fluorouracil), gemcitabine (GEM) combined with capecitabine, or GEM monotherapy.

### Data collection

Clinicopathological data were retrospectively collected from a prospectively maintained database as well as the electronic medical record. The pathological data collected included tumour grade differentiation, resection margins, and the American Joint Committee on Cancer (AJCC) 8th edition stage. Margin determination is based on the “Standardised Pathology Protocol” and the “1 mm” principle [8]. Adverse reactions associated with chemotherapy were assessed using Common Terminology Criteria for Adverse Events Version 5.0 (CTCAE V5.0).

Follow-up was updated until June 30, 2023. Recurrence-free survival (RFS) was evaluated using enhanced CT scans by radiologists and confirmed by experienced specialists in pancreatic tumors. Overall survival data was obtained through telephone interviews.

Our study was approved by the ethics committee of the hospital, and informed consent was obtained from all patients.

### Statistics

Data analysis was performed using the SPSS 26.0 software program and R software version 4.2.0 (http://www.r-project.org/). Normally-distributed continuous variables were presented as mean ± standard deviation and compared using independent t-tests. Non-normally-distributed continuous variables were presented as median (interquartile range) [M(QR)] and compared using the Mann–Whitney U test. Categorical variables were presented as percentages and compared using the χ² test or Fisher’s exact test. Kaplan-Meier analysis was employed to construct the recurrence-free survival (RFS) curve using the R software. Univariate and multivariate RFS survival analyses were conducted using Cox proportional-hazards regression models. The significance level was established at α = 0.05, with *P* < 0.05 denoting statistical significance.

## Results

### Patient characteristics

A total of 220 patients were included in the study, specifically 135 males (61.4%) and 85 females (38.6%), with a median age of 61 ± 9 years. Among them, 126 cases (57.3%) had undergone PD&PPPD surgery, and 94 cases (42.7%) had undergone DP surgery, with a median operation time of 235 (190, 290) minutes and an estimated intraoperative blood loss of 200 (150, 400) ml. The resection margins were as follows: R0 in 95 cases (43.2%), R1 < 1 mm in 88 cases (40.0%), and R1-direct in 14 cases (6.4%). The median postoperative hospital stay was 15 (12, 24) days.

The interval from surgery to the initiation of AC was similar between the AC(d+) and AC(pr) groups (50 vs. 57 days, *P* = 0.108), but significantly shorter in the AC(d+) group compared to the AC(d−) group (50 vs. 61 days, *P* = 0.015). However, patients in the AC(d+) group had a longer interval from surgery to drainage removal compared to those in the AC(d−) group (89 vs. 42 days, *P* < 0.001). Additionally, a smaller proportion of patients in the AC(pr) group underwent distal pancreatectomy compared to those in the AC(d+) group. There were no statistically significant differences in other baseline characteristics, as demonstrated in Table 1.

**Table 1:** Demographics, tumour characteristics, Surgical-related data of AC(d+) and AC(d-) patients

Factor	AC(d+) (*n* = 38)	AC(d-) (*n* = 46)	AC(pr) (*n* = 136)	*P*- value (d + vs. d-)	*P*-value (d + vs. pr)
Age, years, *m ± sd*	60 ± 7	58 ± 10	62 ± 9	0.323	0.158
Sex, *n (%)*				0.569	0.127
Male	27 (71.1)	30 (65.2)	78 (57.4)		
Female	11 (28.9)	16 (34.8)	58 (42.6)		
Preoperative CA 19 − 9 (U/ml), *M(QR)*	111 (47, 404)	140 (44, 473)	129 (53, 299)	0.464	0.688
Operation type, *n (%)*				0.933	0.026
Pancreaticoduodenectomy	17 (44.7)	21 (45.7)	88 (64.7)		
Distal pancreatectomy	21 (55.3)	25 (54.3)	48 (35.3)		
Operation time (min), *M(QR)*	235 (186, 297)	240 (198, 296)	230 (190, 289)	0.951	0.914
Intraoperative blood loss (ml), *M(QR)*	200 (100, 500)	200 (100, 300)	200 (150, 400)	0.901	0.899
Tumor grade differentiation, *n (%)*				0.497	0.688
Well	0	0	2 (1.5)		
Moderate	17 (44.7)	24 (52.2)	54 (39.7)		
Poor	21 (55.3)	22 (47.8)	80 (58.5)		
^#^Resection margins, *n (%)*				0.469	0.612
R0	18 (48.6)	14 (37.8)	63 (51.2)		
R1 < 1 mm	16 (43.2)	17 (45.9)	55 (44.7)		
R1-direct	3 (8.1)	6 (16.2)	5 (4.1)		
^#^T stage, n (%)				0.054	0.330
T1	9 (23.7)	3 (6.5)	15 (12.3)		
T2	12 (31.6)	22 (47.8)	52 (42.6)		
T3	11 (28.9)	18 (39.1)	37(30.3)		
T4	6 (15.8)	3 (6.5)	18 (14.8)		
N stage, n (%)				0.791	0.647
N0	14 (36.8)	19 (41.3)	58 (42.6)		
N1	18 (47.4)	22 (47.8)	53 (39.0)		
N2	6 (15.8)	5 (10.9)	25 (18.4)		
^#^AJCC 8th stage, *n (%)*				0.091	0.298
I	8 (21.1)	9 (19.6)	40 (32.8)		
II	18 (47.7)	31 (67.4)	55 (45.1)		
III	12 (31.6)	6 (13.0)	27 (22.1)		
Causes of inability to remove drainage				0.593	NA
POPF	33 (86.8)	38 (82.6)	NA		
Other causes	5 (13.2)	8 (17.4)	NA		
Postoperative hospital stay (d), *M(QR)*	15 (13, 36)	16 (11, 23)	15 (12, 25)	0.333	0.457
AC regimen				1.000	0.669
Gemcitabine-based	27 (71.1)	32 (69.6)	103 (75.7)		
mFFX-based	8 (21.1)	10 (21.7)	27 (19.9)		
mFFX-gemcitabine combination	3 (7.9)	4 (8.7)	6 (4.4)		
ECOG performance status at initiating AC, *n (%)*				0.279	0.914
0	12 (31.6)	12 (26.1)	48 (35.3)		
1	22 (57.9)	23 (50.0)	72 (52.8)		
2	4 (10.5)	11 (23.9)	16 (11.9)		
Interval from surgery to drainage removal (d), *M(QR)*	89 (68, 111)	42 (33, 55)	14 (8, 23)	< 0.001	< 0.001
Interval from surgery to initiation of AC (d), *M(QR)*	50 (44, 65)	61 (50, 78)	57 (48, 66)	0.015	0.108
Completed six cycles of AC, *n (%)*				0.695	0.452
Yes	31 (81.6)	39 (84.8)	119 (87.5)		
No	7 (18.4)	7 (15.2)	17 (12.5)		

ECOG, Eastern Cooperative Oncology Group; mFFX, mFOLFIRINOX; NA, Not Available; AC, Adjuvant Chemotherapy; ^#^Missing data exist

### Comparison of chemotherapy-related adverse reactions

Of the 220 patients in the study, 107 (48.7%) experienced grade 3–4 chemotherapy-related adverse events, with neutropenia being the most common, occurring in 64 patients (29.1%). Other grade 3–4 adverse events included sensory peripheral neuropathy in 45 patients (20.1%), abdominal infection in 35 patients (15.9%), thrombocytopenia in 29 patients (13.2%), fatigue in 26 patients (11.8%), and anemia in 20 patients (9.1%).

No significant differences in new-onset surgery-related complications, including hemorrhage, ileus, and abdominal infection, were observed between the AC(d+) group and either the AC(d−) or AC(pr) groups. Additionally, no significant differences were observed in the incidence of grade 3–4 chemotherapy-related adverse events. A more detailed description of these adverse events is provided in Table 2.

**Table 2:** Comparison of grade 3–4 Chemotherapy-Related adverse events among different groups

Event	AC(d+) (*n* = 38)	AC(d-) (*n* = 46)	AC(pr) (*n* = 136)	*P*-value (d + vs. d-)	*P*-value (d + vs. pr)
**Hematologic event**					
Anemia	2 (5.3)	4 (8.7)	14 (10.3)	0.685	0.528
Leukopenia	4 (10.5)	5 (10.9)	20 (14.7)	1.000	0.789
Neutropenia	12 (31.6)	14 (30.4)	38 (27.9)	0.910	0.661
Thrombocytopenia	5 (13.2)	4 (8.7)	20 (14.7)	0.725	0.810
Febrile neutropenia	1 (2.6)	4 (8.7)	8 (5.9)	0.372	0.686
**Nonhematologic event**					
Abdominal infection	8 (21.1)	9 (19.6)	18 (13.2)	0.866	0.232
Infection of other sites	4 (10.5)	3 (6.5)	12 (8.8)	0.696	0.754
Hemorrhage	2 (5.3)	1 (2.2)	10 (7.4)	0.587	1.000
Ileus	1 (2.6)	4 (8.7)	8 (5.9)	0.372	0.686
Fatigue	6 (15.8)	4 (8.7)	16 (11.8)	0.337	0.581
Nausea	3 (7.9)	5 (10.9)	9 (6.6)	0.724	0.726
Vomiting	2 (5.3)	5 (10.9)	7 (5.1)	0.449	1.000
Diarrhea	4 (10.5)	3 (6.5)	4 (2.9)	0.696	0.070
Constipation	3 (7.9)	2 (4.3)	7 (5.1)	0.654	0.457
Fever	1 (2.6)	2 (4.3)	14 (10.3)	1.000	0.196
Sensory peripheral neuropathy	10 (26.3)	6 (13.0)	29 (21.3)	0.123	0.514
**Biochemical event**					
ALT/AST increased	1 (2.6)	3 (6.7)	9 (6.6)	0.621	0.693
ALP/γ-GGT increased	2 (5.3)	3 (6.5)	11 (8.1)	1.000	0.736
Blood total bilirubin increased	1 (2.6)	2 (4.3)	8 (5.9)	1.000	0.686
Hypoalbuminemia	0	0	5 (3.7)	/	0.587
Creatinine increased	0	0	0	/	/
APTT prolonged	0	0	0	/	/

ALT, alanine aminotrans-ferase level; AST, aspartate aminotrans-ferase level; ALP, alkaline phosphatase level; γ-GGT, γ-glutamyltransferase level. Activated partial thromboplastin time

### Survival analysis

In June 2023, with a median follow-up duration of 17.0 (14.6, 19.4) months, 101 patients (45.9%) were observed to have local recurrence and/or distant metastasis, and the median RFS was 18.7 (95% CI, 15.3–22.1) months. A total of 43 deaths (19.5%) were observed during the follow-up period, including eight patients in the AC(d−) group, eight patients in the AC(d+) group, and 27 patients in the AC(pr) group. The estimated 1-year and 2-year survival rates were 85.6% and 60.5%, respectively, for the AC(d−) group, and 95.8% and 61.0% for the AC(d+) group. In the AC(pr) group, the corresponding survival rates were 89.1% and 64.0%. The median OS endpoint has not been attained as of the current analysis.

**Fig. 1:**
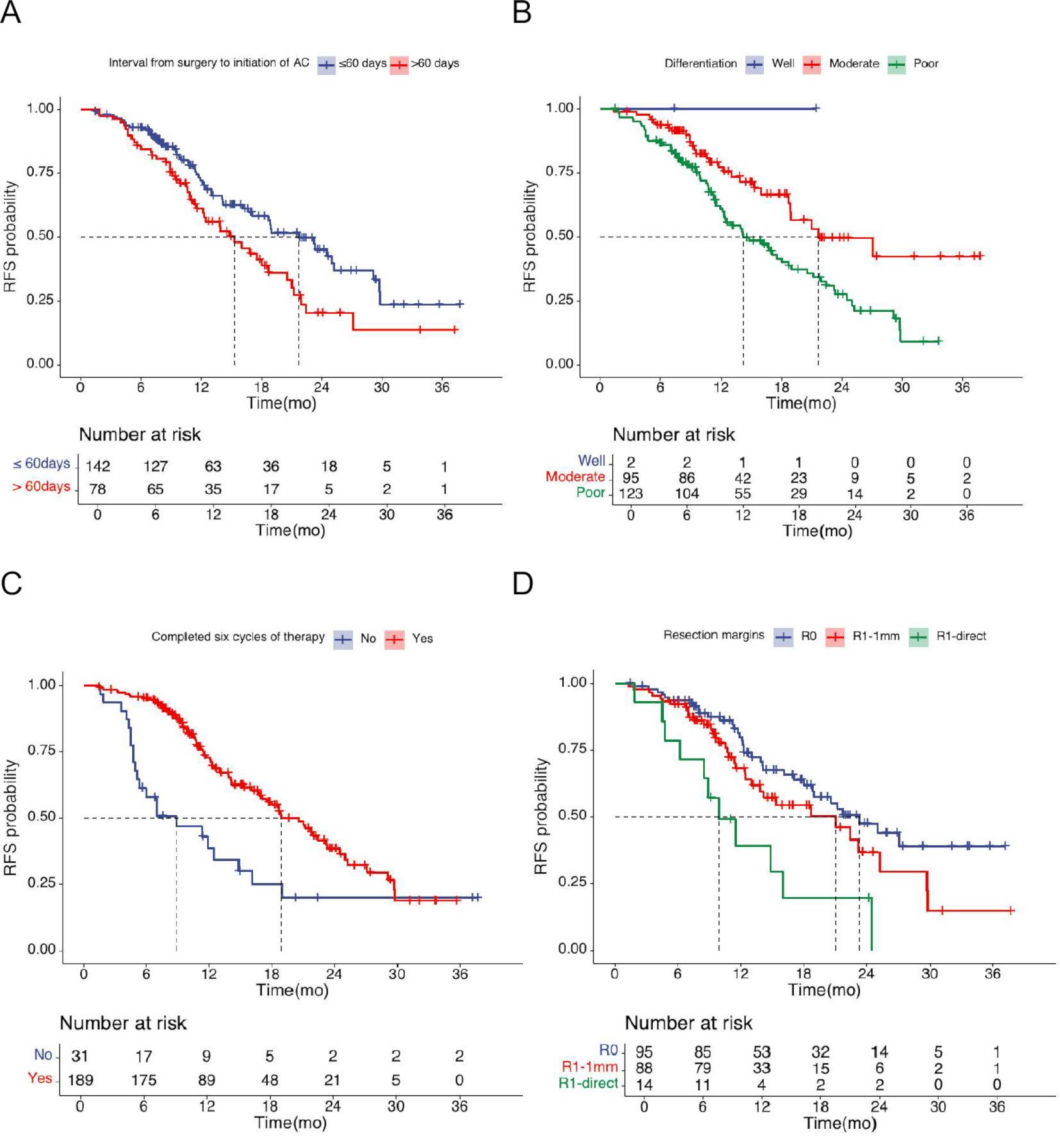
Kaplan Meier plots for RFS by (**A**) Interval from surgery to chemotherapy (< 60 days vs. ≥ 60 days), (**B**) Tumor differentiation (well vs. moderate vs. poor), (**C**) Completed six cycles of chemotherapy (No vs. Yes), (**D**) Resection margin (R0 vs. R1 < 1 mm vs. R1-direct)

Cox univariate regression analysis revealed that the tumour grade differentiation, resection margins, AJCC 8th stage, the interval from surgery to the initiation of AC, and completed six cycles of chemotherapy were significantly associated with RFS (*P* < 0.05). Detailed results are presented in Table 3. Cox multivariate regression analysis unveiled that tumour grade differentiation, completed six cycles of therapy, the interval from surgery to the initiation of AC and resection margins were independent factors affecting RFS. Detailed results are presented in Table 4, and Fig. 1 illustrates the RFS curves.

**Table 3:** Univariate Cox proportional hazards models (*n* = 220)

Factor	No. of Patients	No. of Recurrence	Median RFS Time	HR (95% CI)	*P*-value
Age, years	Continuous variable				0.162
Sex					
Female	135	58	17.5 (10.3–24.7)		
Male	85	43	18.9 (16.5–21.2)	1.126 (0.757–1.674)	0.295
Preoperative CA 19 − 9 (U/ml)	Continuous variable				0.179
Surgery					
Pancreaticoduodenectomy	126	55	21.2 (16.7–25.7)		
Distal pancreatectomy	94	46	16.1 (12.7–19.5)	1.353 (0.908–2.016)	0.137
^*^Tumor grade differentiation					
Moderate	95	28	21.7 (12.5–30.8)		
Poor	123	73	14.2 (10.3–18.1)	2.001 (1.294–3.097)	0.001
^#^Resection margins					
R0	95	35	23.3 (18.0-28.6)		
R1 < 1 mm	88	35	21.0 (12.9–29.1)	1.478 (0.922–2.370)	0.105
R1-direct	14	11	9.9 (6.1–13.7)	3.289 (1.660–6.519)	0.001
^#^AJCC 8th stage					
I	55	20	21.7 (9.7–33.7)		
II	102	47	18.9 (13.3–24.5)	1.433 (0.846–2.424)	0.181
III	47	25	13.0 (10.2–15.9)	2.321(1.280–4.208)	0.006
ECOG performance status at initiating AC					
0	72	36	21.7 (16.8–26.6)		
1	117	46	18.0 (12.8–23.3)	1.291 (0.829–2.008)	0.258
2	31	19	16.0 (11.5–20.5)	1.570 (0.899–2.741)	0.113
AC regimen					
Gemcitabine-based	162	77	18.7 (16.3–20.1)		
mFFX-based	45	17	25.0 (9.1–40.9)	0.859 (0.508–1.455)	0.573
mFFX-gemcitabine combination	13	7	14.1 (13.6–14.5)	1.296 (0.596–2.814)	0.513
AC (d+)					
No	182	85	18.7 (15.7–21.6)		
Yes	38	16	23.2 (11.7–34.7)	0.970 (0.567–1.659)	0.911
Interval from surgery to drainage removal (d)	Continuous variable				0.521
Interval from surgery to initiation of AC (d)	Continuous variable				0.023
Completed six cycles of therapy					
No	31	22	8.9 (2.5–15.3)		
Yes	189	79	18.9 (15.5–22.3)	0.415 (0.258–0.668)	< 0.001

ECOG, Eastern Cooperative Oncology Group; mFFX, FOLFIRINOX; AC, Adjuvant Chemotherapy; ^*^Two well-differentiated tumors were excluded from analysis; ^#^Missing data exist

**Table 4:** Multivariate Cox proportional hazards models (*n* = 220)

Factor	SE	Wald	HR(95% CI)	*P*-value
Interval from surgery to initiation of AC	0.005	4.916	1.012 (1.001–1.023)	0.027
Tumor grade differentiation	0.269	11.690	2.510 (1.481–4.254)	0.001
Resection margin	0.175	10.032	1.740 (1.235–2.451)	0.002
AJCC 8th stage	0.178	2.932	1.356 (0.957–1.922)	0.087
Completed six cycles of therapy	0.292	7.771	0.443 (0.250–0.785)	0.005

## Discussion

Despite a relatively high overall incidence of complications following pancreatic surgery, advances in the management and understanding of complications have led to significant decreases in mortality and reoperation rates,^9^, ^10^]. enabling the possibility of administering AC without the removal of intraperitoneal drainage. Our findings, for the first time, indicate that there was no increase in the incidence of grade 3–4 adverse reactions and new-onset surgery-related complications associated with AC(d+) treatment. These preliminary results suggest that AC(d+) is a safe and feasible treatment approach for patients who are unable to remove intraperitoneal drainage following pancreatic cancer resection.

Before proceeding with AC(d+), careful consideration ought to be given to two critical factors. Firstly, the patient’s physical and nutritional status should be assessed. Patients with a poor physical or nutritional status are typically advised to receive enteral nutrition either in the hospital or at home. Secondly, the possibility of obstructed abdominal fluid drainage should be ruled out, with imaging examinations serving as the primary method of assessment. In cases where fluid accumulation is detected around the drainage tube, it may be necessary to either replace the tube or withdraw it by 1–2 cm. Conversely, if the fluid accumulation is remote from the drainage tube, percutaneous puncture drainage may be considered.

The incidence of granulocytopenia following AC for pancreatic cancer ranges from 23.0 to 77.8%, with granulocyte deficiency occurring in approximately 5.4–22.2% of cases [11, 12, 13, 14]. When combined with infection, this condition carries a high mortality rate. To prevent granulocyte deficiency in high-risk patients (e.g. those with low baseline white blood cell counts, liver dysfunction, or high ECOG scores) before AC(d+), proactive measures such as nutritional support and prophylactic use of G-CSF should be considered. In the case of granulocyte deficiency and infection, blood and drainage cultures for bacteria ought to be performed, followed by the administration of broad-spectrum antibiotics and G-CSF.

Interestingly, chemotherapy drugs have the potential to induce the degeneration of pancreatic cells, widespread lobular atrophy, tissue fibrosis, and diminished exocrine pancreatic function, which could potentially lead to a decrease in the secretion of pancreatic fluid [15, 16, 17]. This effect might well be influenced by the specific chemotherapy regimen. 5-Fu has demonstrated efficacy in treating acute pancreatitis and refractory pancreatic fistula,^18^, ^19^, ^20^]. whilst gemcitabine-based regimen do not have this effect. In this study, AC(d+) did not shorten the duration of drainage. The interval from surgery to drainage removal was significantly longer in the AC(d+) group compared to the AC(d−) group (89 days vs. 42 days, *P* < 0.001). The high proportion (70.2%) of gemcitabine-based regimens used in the AC(d+) group may have contributed to this finding. Further analysis revealed that in the AC(d+) group, the duration of drainage for patients receiving the mFOLFIRINOX-based regimen was notably shorter than for those receiving the gemcitabine-based regimen (55 days vs. 64 days, *P* = 0.062), although this difference did not reach statistical significance. Additionally, existing literature suggests that chemotherapy may impair the healing of fistulas, which could be an important factor contributing to prolonged drainage time [21]. To determine which factor plays a more significant role, additional large-scale controlled trials are needed to validate these findings.

In the CONKO-001 study, chemotherapy was initiated between days 10 and 42 postoperatively [22]. Shortly thereafter, the ESPAC-3 and ESPAC-4 trials initiated chemotherapy within 12 weeks based on expert consensus [23, 24]. Consequently, the NCCN guidelines suggest initiating AC within 12 weeks after adequate recovery from surgery [7]. However, some patients may experience delayed initiation of AC beyond 12 weeks due to the inability to remove the abdominal drainage.

Moreover, despite current guidelines recommending AC initiation within 12 weeks after surgery, several studies have reported a poorer prognosis in patients with delayed initiation of AC within this timeframe [25, 26]. Sung Jun et al [25]. found that patients with stage I–II pancreatic cancer who commenced adjuvant therapy 28–59 days following primary surgical resection exhibited a superior 2-year survival rate (51.3% vs. 45.4%, *P* = 0.01) and OS (22.4 vs. 20.4 months, *P* = 0.01) in comparison with those who initiated adjuvant treatment before 28 days or after 59 days. In a study conducted by Richard et al.,^26^]. propensity score matching analysis revealed a significant association between earlier delivery (< 66 days) of AC and improved survival in patients with stage I–III pancreatic cancer. The 5-year survival rate was 20% in patients who received AC within 66 days in comparison to 18% in those who did not (*P* = 0.0266).

For patients with a high risk of recurrence, early administration of chemotherapy may offer potential survival benefits [5]. The data from the ESPAC-3 study indicated that the ability to complete six cycles of AC is an independent prognostic factor for survival in pancreatic cancer, which aligns with our findings. However, in that study, for patients unable to complete all six cycles of chemotherapy, the timing of chemotherapy initiation emerged as an independent factor influencing both disease-free survival (DFS) and overall survival (OS) [3]. Recent studies have also found that, for pancreatic cancer patients who are positive for postoperative circulating tumour cells, delaying chemotherapy significantly shortens the time to RFS (12.4 vs. 17.9 months, *P* = 0.004).^5^ Moreover, despite the controversy surrounding the issue, several studies have suggested that POPF is a significant risk factor for high recurrence rates in patients with pancreatic cancer [16, 27, 28, 29, 30, 31]. A meta-analysis conducted by Grego et al. (2021) reported a 59% increased risk of tumour recurrence in patients with pancreatic cancer who developed POPF [29]. Likewise, Nagai et al [28]. discovered that pancreatic fistula after surgery was associated with a four times higher risk of peritoneal recurrence. Multivariate analysis in this study demonstrated that the interval from surgery to the initiation of AC is an independent factor affecting RFS. AC(d+) may be more warranted in this particular patient population.

Additionally, Our study identified tumor differentiation and resection margin status as independent prognostic factors for survival, consistent with previous literature. Well-differentiated tumors typically indicate less aggressive biology and better outcomes, while negative resection margins reflect more complete tumor clearance and improved prognosis. Carlo Ingaldi et al. reported that well-differentiated tumors (G1), R0 resections, and adjuvant chemotherapy were all associated with longer survival (*p* = 0.010, 0.019, and 0.052, respectively) [32]. Similarly, Yamamoto et al. identified R0 resection as an independent favorable prognostic factor (HR = 0.48; 95% CI: 0.30–0.77; *P* = 0.003) [33]. Luu AM et al. also confirmed tumor grade as an independent predictor of survival in PDAC, underscoring its value in postoperative risk stratification [34].

There exist certain limitations in this study. Firstly, our study was constrained by a limited sample size and a retrospective design. Although the baseline characteristics of the two groups are comparable, there remains a potential for residual confounding. Secondly, our study preliminarily demonstrates the safety and feasibility of AC(d+), while analyzing the factors influencing RFS. However, a long-term survival analysis is still required, and extended follow-up is essential to ascertain enduring survival outcomes.

## Conclusion

Administering AC was safe for patients who underwent resection for PDAC and encountered challenges in the prompt removal of intraperitoneal drainage beyond 30 days post-surgery. The proactive management of preventing delays in chemotherapy administration could reduce the early recurrence risk in this particular patient cohort.

## Data Availability

The datasets used and/or analysed during the current study are available from the corresponding author on reasonable request.
